# Antibiotics and Antibiotic Resistance Genes in Animal Manure – Consequences of Its Application in Agriculture

**DOI:** 10.3389/fmicb.2021.610656

**Published:** 2021-03-29

**Authors:** Magdalena Zalewska, Aleksandra Błażejewska, Agnieszka Czapko, Magdalena Popowska

**Affiliations:** Department of Bacterial Physiology, Institute of Microbiology, Faculty of Biology, University of Warsaw, Warsaw, Poland

**Keywords:** antibiotic resistance genes, antibiotic-resistant bacteria, antibiotic use, animal agriculture, fecal matter, manure resistome, soil resistome, plant resistome

## Abstract

Antibiotic resistance genes (ARGs) are a relatively new type of pollutant. The rise in antibiotic resistance observed recently is closely correlated with the uncontrolled and widespread use of antibiotics in agriculture and the treatment of humans and animals. Resistant bacteria have been identified in soil, animal feces, animal housing (e.g., pens, barns, or pastures), the areas around farms, manure storage facilities, and the guts of farm animals. The selection pressure caused by the irrational use of antibiotics in animal production sectors not only promotes the survival of existing antibiotic-resistant bacteria but also the development of new resistant forms. One of the most critical hot-spots related to the development and dissemination of ARGs is livestock and poultry production. Manure is widely used as a fertilizer thanks to its rich nutrient and organic matter content. However, research indicates that its application may pose a severe threat to human and animal health by facilitating the dissemination of ARGs to arable soil and edible crops. This review examines the pathogens, potentially pathogenic microorganisms and ARGs which may be found in animal manure, and evaluates their effect on human health through their exposure to soil and plant resistomes. It takes a broader view than previous studies of this topic, discussing recent data on antibiotic use in farm animals and the effect of these practices on the composition of animal manure; it also examines how fertilization with animal manure may alter soil and crop microbiomes, and proposes the drivers of such changes and their consequences for human health.

## Antibiotic Use in Animal Farms

The spread of antibiotic-resistant bacteria (ARB) is a growing problem worldwide. In 2017, the most important ARB were included in a list published by the World Health Organization (WHO). The ARB families that were believed to pose the greatest threat to human health were categorized as critical, high or medium priority, depending on the need to seek effective treatment options.

It has been estimated that antimicrobial resistance (AMR) is responsible for 25,000 deaths/year in the European Union (EU), and 700,000 worldwide. It has also been predicted that by 2050, AMR will be responsible for more deaths than cancer (European Commission, 2018). With the discovery of penicillin, in 1928, many life-threatening or even lethal diseases became curable, with clear benefits for veterinarians and animal breeders; however, since the 1960s, antibiotics have been widely applied in sub-lethal doses as growth promoters for food-producing animals (Hassan et al., 2018). Such extensive, uncontrolled use may result in the presence of low, sub-inhibitory concentrations in the tissues and guts of treated animals (Lim et al., 2020) and in the environment (Khan et al., 2013). Although the precise complete mechanisms of action remain unclear, it has been hypothesized that when applied in sub-lethal doses, antibiotics stimulate the intestinal synthesis of vitamins, lower the total amount of bacteria in the intestinal tract by reducing competition between microorganism and host for nutrients, inhibits the growth of harmful bacteria, and modifies the microbial metabolism of the rumen (Economou and Gousia, 2015).

However, the chronic application of such sub-therapeutic doses favors the selection of ARB by promoting their growth or introducing *de novo* mutations. One of the first reviews of the effect of sublethal doses of antibiotics on the bacterial community was published by Lorian (1975). The presence of ARB may enhance the transfer of antibiotic-resistant genes (ARGs) between enteric bacteria in the intestinal tract of the host animal, and affect quorum sensing (e.g., azithromycin in *Pseudomonas aeruginosa*, *Staphylococcus aureus*, or *Streptococcus pneumoniae*). In addition, the presence of antibiotics can stimulate biofilm formation and horizontal gene transfer (HGT) in some bacteria; for example, the transfer of azithromycin, ciprofloxacin or tigecycline resistance has been observed in *Enterococcus faecalis* and *P. aeruginosa*. It can also increase the rate of recombination and selected gene expression in the bacterial community, such as ciprofloxacin, norfloxacin, rifampicin, gentamycin, and tetracycline in *Escherichia coli*, *P. aeruginosa*, or *S. aureus* (Lorian, 1975). The mechanisms of action of sublethal levels of antibiotics on bacteria are described in detail by Andersson and Hughes (2014). In food-producing animals, antibiotic use influences the functions of enteric bacteria and can temporarily increase antibiotic resistance in the fecal microbiome (Chee-Sanford et al., 2009; Broom, 2017).

**FIGURE 1:**
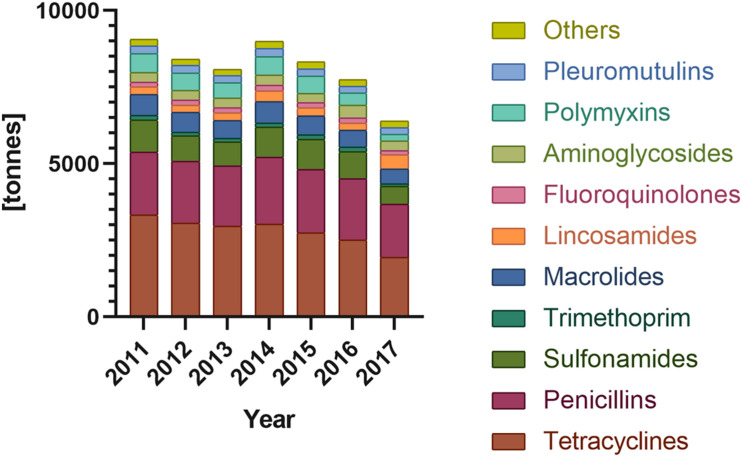
Antibiotic sales for food-producing animals in Europe sort by antibiotic classes (according to ECDC/EFSA/EMA second joint report on the integrated analysis of the consumption of antimicrobial agents and occurrence of antimicrobial resistance in bacteria from humans and food-producing animals, 2017).

**FIGURE 2:**
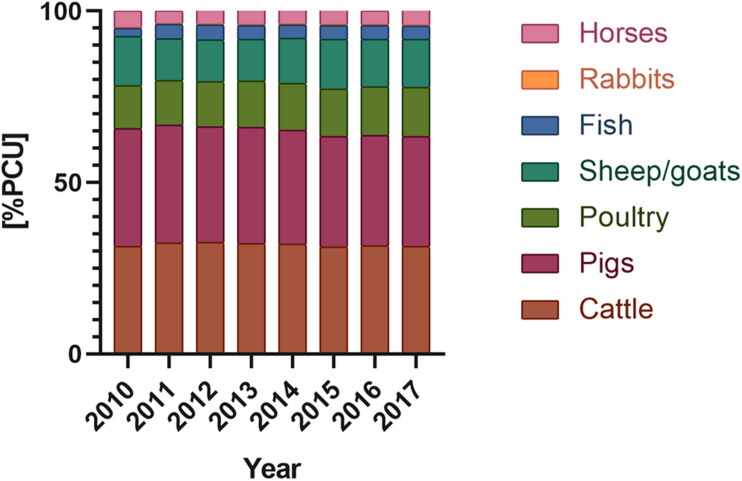
Antimicrobials used in domestic animals (according to according to ECDC/EFSA/EMA second joint report on the integrated analysis of the consumption of antimicrobial agents and occurrence of antimicrobial resistance in bacteria from humans and food-producing animals, 2017) [PCU] – population corrected unit; PCU a technical unit of measurement, used only to estimate sales corrected by the animal population in the individual countries; 1 PCU = 1 kg of different categories of livestock and slaughtered animals.

The majority of ARG transmission occurs *via* HGT, i.e., whereby mobile genetic elements (MGE), such as plasmids or transposons coding for ARGs, are exchanged between bacterial species, even those that are not closely related (Redondo-Salvo et al., 2020). The dynamics of transfer depend not only on the presence of positive selection, i.e., antibiotic concentrations in the environment ranging from values much higher than minimal inhibitory concentrations to those several hundred-fold lower, but also the spatial structure of the community and the presence of predators (Cairns et al., 2018). The WHO, World Organization for Animal Health (OIE), and the Food and Agriculture Organization (FAO) have officially stated that the non-human use of different types of antimicrobials may have harmful consequences for human health (So et al., 2015). Although some countries have officially restricted the use of antimicrobials in livestock to only medical purposes (e.g., the EU in 2006 in accordance to 1831/2003/EC legislation), they are still overused on some regions characterized by highly-intensive livestock production, such as the United States, Russia, India, China, and South Africa. In the US, antimicrobial treatment in food-producing animals has been estimated to account for approximately 80% of total annual use; the vast majority of these antimicrobials are believed to include essential human medicines used for the treatment of common infections, or which are necessary for performing surgeries, organ transplantations or chemotherapy in humans (Van Boeckel et al., 2015) (Figures 1, 2).

A study of antimicrobial use in Southeast Asia indicated that, excluding feed, farmers in this region administered 46 mg of different antimicrobial compounds per kg of live pigs and 52–276 mg per kilogram of live chickens per year (Nhung et al., 2016). In addition, a 2014 study in the EU found the average intake of antimicrobials to be 123.7 mg/kg in humans and 151.5 mg/kg in animals (European Centre for Disease Prevention, and Control [ECDC], European Food Safety Authority [EFSA], and European Medicines Agency [EMA], 2017).

The use of antibiotics for non-medical purposes, or for treating entire extensive animal production facilities when a single animal falls ill, has resulted in a growth in ARB in the animal production chain. It has been estimated that 84% of the antimicrobial compounds administered in chicken farms in the Mekong Delta region were given only for prophylactic purposes; of these, the most common were penicillins, lincosamides, quinolones, and combinations of sulfonamides with trimethoprim (Nhung et al., 2016). Elsewhere, the most common antibiotics found to be present in pig, beef and turkey manure were tetracyclines (oxytetracycline and chlortetracycline), tylosin, sulfamethazine, amprolium, monensin, virginiamycin, penicillin, and nicarbazin (De Liguoro et al., 2003; Kumar et al., 2005).

The Food and Drug Administration (FDA) has approved 18 classes of antimicrobials for use in food-producing animals (Medicine, 2019). However, the WHO reports that 57% of all antimicrobials used in animal production are essential for human medicine, including amoxicillin, colistin, tetracyclines, neomycin, lincomycin, and bacitracin. Of the FDA classes, the most widely used in human medicine worldwide are the penicillins, macrolides, and fluoroquinolones, while the tetracyclines, penicillins, and sulfonamides were the highest selling for food-producing animals.

In the EU/EEA (the European Union/European Economic Area) carbapenems and monobactams are not approved for use in food-producing animals, and pleuromutilins are not authorized for use in humans. In addition, higher total consumption of the penicillins, cephalosporins (all generations) and fluoroquinolones was demonstrated by humans than food-producing animals (European Centre for Disease Prevention, and Control [ECDC], European Food Safety Authority [EFSA], and European Medicines Agency [EMA], 2017). A complete list of antibiotics allowed for use in food production animals, divided according to veterinary importance (Critically Important, Highly Important and Important) was first prepared in 2007 by the OIE in consultation with the FAO and WHO, and is constantly being revised; the most recent update was released in May 2018 by the World Assembly of OIE Delegates (International Office of Epizootics, 2015; Góchez et al., 2019).

Although environmental factors have long been known to affect human health, the relationship between the spread of AMR and antibiotic use in agriculture was first recorded in the 1969 Swann report (Soulsby, 2007). Even though antibiotics and ARGs/ARB are ancient and naturally-occurring substances, they are nevertheless considered emerging pollutants associated closely with human-impacted environments (He et al., 2019; Zhang et al., 2019). The global debate on the scale of the threat posed by antimicrobial use in livestock was intensified by the recent finding of a plasmid-mediated colistin resistance gene (*mcr-1*) in commensal *E. coli* from pigs, pork products and humans in China (Liu et al., 2016).

In 2008, the FAO, WHO, OIE, the United Nations Children’s Fund (UNICEF), United Nations System Influenza Coordination, and the World Bank developed a holistic approach entitled “Contributing to One World, One Health-A Strategic Framework for Reducing Risks of Infectious Diseases at the Animal-Human-Ecosystems Interface” including a recommendation for a One Health action plan for global health. In 2017, the European Commission adopted the WHO One Health framework, naming AMR as one of the main concerns. The proposed approach holistically regards the health of humans, animals, and different environmental compartments, such as water or soil, as a single network, where one part inseparably depends on another. This framework has three major aims: (1) making the EU a region with best practice in AMR management, (2) boosting research, development, and innovation in AMR, and (3) shaping the global approach to addressing AMR. Each aim has been divided into smaller tasks that address existing gaps in knowledge concerning AMR in the environment, such as the role of the environment in ARGs/ARB transmission, the routes of ARGs/ARB transmission, the most critical hot spots, human health risk assessment, and the establishment of tracking and detection systems.

The current limitations on antimicrobial use in livestock are explained in the document “Council conclusions on the next steps under a One Health approach to combat AMR” (2016). In addition, a good review of the existing global policies on antibiotics use in livestock is provided by Walia et al. (2019).

## The Use Of Antibiotics In The Poultry Industry And Its Effect On The Poultry Manure Resistome

One of the most significant sectors of meat and egg production worldwide is that of poultry production. In 2018, global poultry population (laying hens and broilers) had reached 23.7 billion, growing from 22.85 billion in 2017 and 14.38 billion in 2000. The largest chicken producer in 2018 was China, with 5274.48 million animals, followed by Indonesia with 2384.15 million, and the US with 1973.38 million. China is also the largest egg producer globally, with 529 billion eggs, followed by the US with 106.7 billion. Moreover, in 2018, in the US alone, 22.6 million tons of broiler meat was produced. It has been predicted that in 2020, the global export of poultry meat will increase by 3.7% to reach 13.8 million tones. Thailand, Brazil, Turkey, Ukraine, the US, and the EU are the leading poultry exporters, from whom almost 80% of exported poultry meat originates (FAOSTAT, 2020).

Since the discovery of antibiotics, strict biosecurity guidelines and prevention techniques have been employed by the poultry and meat production industry to not only increase its production efficiency and eliminate the risk of zoonotic diseases, but enhance the growth and weight of the broilers (Collett et al., 2019). Although the use of antibiotics is strongly regulated in food-producing animals in the EU and the US (European Commission, 2005; World Health Organization and WHO Advisory Group on Integrated Surveillance of Antimicrobial Resistance (AGISAR), 2017), they are often used for disease prevention in many poultry exporting countries, such as Brazil and China (Roth et al., 2019). A few classes of antibiotics are approved as additions to animal fodder in Canada, such as bambermycin, bacitracin, salinomycin, penicillin, virginiamycin, and chlortetracycline (Diarra and Malouin, 2014).

In addition, in a 2017 study of eight farms in Thailand, where 14,000 3 kg broilers were raised, it was estimate that 303 mg of antibiotics were used per chicken as routine prophylaxis; this mixture included amoxicillin, colistin, doxycycline, oxytetracycline, and tilmicosin. According to the WHO, amoxicillin and colistin are critically important for human medicine, with colistin being considered the primary therapeutic option to treat diseases caused by resistant gram-negative bacteria, particularly enterobacteria (Wongsuvan et al., 2018).

In survey-based studies in Ghana in 2016, farmers from 400 poultry farms reported using antibiotics for prophylactic purposes, including penicillins (26.22%), tetracyclines (24.13%), and aminoglycosides (20.51%) (Andoh et al., 2016).

Besides growth promotion and disease prevention, antibiotics are also prescribed by veterinarians for treating illnesses. In 2015, a total of 8361 tons of antimicrobial agents were sold for veterinary use in the EU (European Medicines Agency, 2017).

Tetracyclines, with their broad spectrum of action, are one of the most frequently-used antimicrobials in the poultry industry, not only for prophylaxis or antibacterial therapy but also as growth promoters (Ljubojević et al., 2017). Interestingly, while tetracyclines constitute two-thirds of the antibiotics applied to animals in the US (Gonzalez Ronquillo and Angeles Hernandez, 2017), they accounted for less than 40% in the EU between 2015 and 2017 (OIE, 2018). However, the tetracyclines are poorly absorbed by the animal, and their active residues can concentrate in meat, urine, and feces. A study of broiler farms in Egypt, found high levels of chlortetracycline and oxytetracycline in chicken litter and droppings, these values being, respectively, 6.05 and 2.47 μg/g for chlortetracycline and 5.9 and 1.33 μg/g for oxytetracycline. Slightly lower amounts of tetracycline and doxycycline were found: 1.9 μg/g in litter and 0.46 μg/g in droppings for tetracycline, and 0.87 μg/g and 0.02 μg/g for doxycycline. More importantly, the researchers report the presence of three tetracycline resistance genes (*tetM*, *tetW*, *tetQ*) in the intestinal contents: *tetW* was observed in all analyzed samples, *tetQ* in 42% of them, and *tetM* in only 17% (Mahmoud and Abdel-Mohsein, 2019).

Despite being banned as growth promoters in the early 1970s (EC directive 70/524), the total consumption of tetracyclines in food-producing animals in Europe was still estimated at 3138 tons in 2012. Carballo et al. (2016) report that oxytetracycline was present in 37% of tested manure samples, with a maximum concentration of 0.88 mg/kg, and doxycycline in one sample, with a concentration of 0.53 mg/kg. The existence of antibiotic residues in the environment creates on-site selection pressure, may result in the presence of ARB, which are often isolated from manure samples. In Nigeria, five potential human pathogens were isolated from chicken manure, such as *Salmonella typhi*, *E. coli*, *Shigella dysenteriae*, *S. aureus*, and *Aeromonas hydrophila*, all of which were resistant to tetracycline (Omojowo and Omojasola, 2013).

Arguably, a greater threat to human and animal health is posed by multidrug-resistant (MDR) bacteria, with an alarming increase being observed in the number of MDR strains isolated from the poultry meat production chain (Afridi et al., 2020). Unusually high levels of MDR bacteria, especially those of the *Enterobacteriaceae*, have been isolated from poultry. Portuguese studies based on livestock manure taken from pig, dairy and poultry farms and slaughterhouses found the highest level of resistance to tetracycline, trimethoprim/sulfamethoxazole, chloramphenicol, and amoxicillin/clavulanic acid in strains isolated from poultry farms. In addition, the highest prevalence of ciprofloxacin-resistant bacteria was observed in poultry, which may be related to the growing use of ciprofloxacin to treat infections. In addition, although MDR bacteria were isolated from all samples, the highest number of strains resistant to seven or more antibiotics were isolated from poultry samples. A number of chloramphenicol, quinolone, tetracycline, and sulfonamide resistance genes were identified (Amador et al., 2019).

A similar trend was also noted in Belgium. A study of AMR in commensal *E. coli* isolated from veal calves, young beef cattle, broiler chickens, and slaughtered pigs found the highest number of isolated MDR bacteria to be present in broiler chickens (Hanon et al., 2015).

A high prevalence of *E. coli* and *Klebsiella pneumoniae* resistant to β-lactams with ESBL (Extended-Spectrum Beta-Lactamase) and AmpC (clinically important β-lactamases; cephalosporinases) phenotypes was also identified in a study of MDR gram-negative bacteria isolated from poultry feces kept on farms in the Lebanon. The real-time PCR assay performed on 112 strains of ESBL producers detected *bla*_SHV_ in 20% of strains, *bla*_TEM_ in 89%, and *bla*_CTX–M_ in 53%. In addition, more than half (66%) of the isolated ESBL/AmpC strains were also resistant to gentamicin, but only one AmpC β-lactamase gene (*bla*_CMY_) was found among all samples (Dandachi et al., 2018).

Resistance to extended spectrum cephalosporins (ESBL genes) has also been observed among *Salmonella* serovars. This could have particularly serious consequences, as these bacteria are key etiological factors in diseases among poultry, and may also cause severe illness in humans; the acquisition of antibiotic resistance may significantly limit the range of treatment options (Büdel et al., 2020). Rayamajhi et al. (2010) report the presence of ampicillin-resistant *Salmonella* isolates in poultry feces, the environment and egg yolks on poultry farms in South Korea (Table 1). Among all antibiotics tested, resistance was most commonly observed for sulfamethoxazole (74.7%) and nalidixic acid (63.7%), with streptomycin (38.5%), tetracycline (28.6%), and amoxicillin resistance (18.7%) being less common. Furthermore, the *bla_TEM–1_* gene was present in all ampicillin-resistant strains and the *bla_DHA–1_* gene in stains with lower susceptibility to cefoxitin, as indicated by β-lactamase production ability. A 2019 study from Kenya found that almost 50% of *Salmonella* samples isolated from manure from broilers and layers were resistant to amoxicillin. In addition, 28% of *Salmonella* strains were resistant to co-trimoxazole, 11%, to tetracycline and 6% to streptomycin. A search for ARGs revealed the presence of *bla*_TEM_ in 46% of all isolated bacteria with the *bla*_CTX–M_ gene present in 18% (Langata et al., 2019).

**TABLE 1:** Antibiotic resistance bacteria and genes in poultry manure.

Bacteria species	Phenotype	Resistance genes	Source	Country	References
**Poultry manure**					
*Salmonella* spp.	Extended spectrum cephalosporins	*bla*_TEM–1_, *bla*_DHA–1_	Feces, eggshell, dead egg yolk, cloaca, liver, water, environmental dust	South Korea	Rayamajhi et al., 2010
*Escherichia coli*		*bla*_TEM_, *bla*_SHV_, *bla*_CTX–M_	Fecal swabs	Lebanon	Dandachi et al., 2018
*Klebsiella* spp.					
*Enterobacteriaceae*	Quinolones (CIP)	*qnrB, qnrC, qnrD, qnrS*	Poultry manure	Portugal	Amador et al., 2019
	Tetracyclines (TET)	*tetA, tetB, tetC, tetE, tetK, tetL, tetM, tetO*			
	Sulfonamides	*sul1, sul2, sul3*			
*Enterococcus* spp.	Vancomycin (VRE)	*vanA*	Poultry manure	Greece	Tzavaras et al., 2012
*Enterococcus* spp.	Macrolides (Erythromycin)	*ermB, ermA, mrsC*	Poultry manure	United States	Graham et al., 2009
*Staphylococcus* spp.	Macrolides (Erythromycin)	*ermA, mrsA/B*			
*Staphylococcus* spp.	MRSA	*mecA; mecR1; mecI*	Poultry manure	South Korea	Lee, 2006

Together with the high prevalence of the MDR strains among the *Enterobacteriaceae* family, the spread of Vancomycin-Resistant Enterococci (VRE) is widely regarded as one of the greatest threats to human health care. A key factor in the spread of vancomycin resistance is believed to involve a glycopeptide antibiotic called avoparcin, which has been applied in animal husbandry as a growth promoter. Although its use in livestock was banned in the EU early in 1997 (EC directive 97/6/EC), relatively high numbers of VRE are still observed in poultry meat production in the EU, and this can be considered a potential reservoir of vancomycin resistance. One study in Greece found that approximately 14.4% of the *E. faecalis* strains isolated from broilers and poultry fecal samples showed vancomycin resistance, and isolated strains demonstrated resistance to several other antibiotics, such as tetracycline (100%), erythromycin (54.4%), ampicillin (16.5%), and ciprofloxacin (30.4%). Analyses have detected the *vanA* gene in 14.4% of fecal samples from poultry and the *vanC* gene in 8.2% (Table 1) (Tzavaras et al., 2012).

Together with the emergence of VRE strains, the preservation of resistant gram-positive bacteria in stored poultry manure is also of great concern. One study by Graham et al. (2009) examined the fate of antibiotic resistant *Enterococcus* spp. and *Staphylococcus* spp. and the persistence of ARGs in litter after a 120-day storage period (Table 1). The findings revealed the presence of quinupristin-dalfopristin resistance in 11% of the isolated *Staphylococcus* strains and erythromycin resistance in 57%; in addition, 31% of *E. faecium* were resistant to both antibiotics. The most commonly identified ARGs were *ermB* in *Enterococcus* spp. and *ermA* in *Staphylococcus* spp. Moreover, the *msrC* gene was found in one *Enterococcus* isolate and this was found to be homologous with *msrA* found in *Staphylococcus* spp. It should be stressed that ARGs were found in all collected samples during a 120-day storage period or even later (Graham et al., 2009).

*Staphylococcus* spp. are considered typical of the chicken microflora. Although they are harmless in most cases, they are known to act as opportunistic pathogens, and can cause severe infections, particularly the bacteria that harbor ARGs. Methicillin-resistant *S. aureus* (MRSA) isolated from poultry may pose a considerable risk for human health, as a high chance of zoonotic transfer exists during the breeding or fattening period (Zaheer et al., 2017). MRSA bacteria were found to be present in almost 90% of fecal chicken swabs taken from small poultry farms in Nigeria, and more worryingly, in 83.3% of nasal swabs taken from personnel working on the farms (Journals and Oke, 2013). MRSA bacteria harboring *mecA* gene were also successfully isolated from chicken samples in South Korea (Lee, 2006) (Table 1).

Subtherapeutic doses of antibiotics used in poultry farms exert selection pressure on the bacteria of the intestinal flora of the animals, thus encouraging the spread of ARB into the environment (Suresh et al., 2018). Being located on MGEs such as plasmids, transposons and integrons, ARGs can easily be transmitted between bacteria by HGT. A good example of such spread is that of plasmid-mediated colistin resistance; this can represent a considerable risk for human health, as colistin is one of the strategic drugs used against carbapenem-resistant *Enterobacteriaceae* (Wang et al., 2018). Unfortunately, because of the long history of colistin usage in animals and the complete lack of surveillance until 2014 (Commission Implementing Decision 2013/652/EU), it is difficult to track colistin usage and detect resistance (Kempf et al., 2016). However, what is certain is that the first plasmid carrying the colistin resistance gene *mcr-1* was isolated from *Enterobacteriaceae* bacteria from a pig fattening facility (Liu et al., 2016). In Brazil, of the 280 *Enterobacteriaceae* strains isolated from chicken fecal samples in a study from 2003 to 2015, 113 were found to be colistin resistant; the *mcr-1* gene was identified in 14 *E. coli* strains (Fernandes et al., 2016). Elsewhere, the *mcr-1* gene was detected in eight of 12 (∼66%) tested chicken manure samples (Gao et al., 2019); interestingly, the presence of detected *mcr-1* genes was effectively reduced by 90% during a 30-day composting process.

Class 1 and 2 integrons are also commonly found among MDR *Enterobacteriaceae* isolates, which may indicate that resistance is mediated by plasmid transfer. A study of the *int1* and *int*2 genes in clinical and livestock samples, including poultry, by Goldstein et al. (2001) found the *int1* gene to be present in 79% of *Salmonella* isolates sampled from chickens and *int2* in 2%; however, the *int2* gene was present in only 14% of *E. coli* isolates. Furthermore, a study of 55 *E. coli* strains in broilers and layers in Kenya detected *int1* genes in 26% of isolates, with most coming from β-lactamase positive isolates (Langata et al., 2019).

A full understanding of the development and transmission of AMR in poultry production is essential for creating effective risk management strategies for preventing the spread of resistant bacteria from animals to humans and providing safe sources of food.

## The Use Of Antibiotics In The Cattle Industry And Its Effect On The Cattle Manure Resistome

Global bovine meat production reached 71.1 million tons in 2018, with the largest producers being Brazil, the US, Argentina, the EU, and Australia (FAOSTAT, 2020; Meat Market Review). In addition, there are more than 264 million dairy cows worldwide, producing nearly 600 million tons of milk every year. India has the highest number of dairy cows globally, with over 40 million cows, followed by the US, China, Pakistan, and Brazil (FAOSTAT).

The use of antibiotics in dairy cattle has the potential to stimulate the development and dissemination of ARGs among different types of enteric bacteria characteristic for ruminants, as well as potentially pathogenic species related to the fecal microbiome. Some of these bacteria may be pathogenic to humans, and hence could pose a threat to human health if they additionally acquire ARGs. Moreover, non-pathogenic bacteria from manure might transfer ARGs to pathogens existing in manure, soil, or food consumed by animals or humans (Karami et al., 2007; Brichta-Harhay et al., 2011). Furthermore, manure from dairy cows, commonly used as a soil fertilizer, may harbor diverse new variants of ARGs from the gut microbiota of cattle (Wichmann et al., 2014).

A range of antibiotics are used in the cattle production industry, including aminoglycosides, β-lactams, chloramphenicol, fluoroquinolones, glycolipids, ionophores, macrolides, quinolones, streptogramins, sulfonamides, and tetracyclines. The majority are used in dairy cow husbandry for growth promotion (when allowed), prophylaxis, and treatment of most complex diseases such as mastitis or metritis, or as dry cow therapy: the prophylactic use of antibiotics to avoid mammary gland inflammation in animals after lactation (International Office of Epizootics, 2015). In North America, the major antibiotics administered sub-therapeutically in the diet to beef and dairy cattle are chlortetracycline (16%), tylosin (10%), and sulfamethoxazole (9%). These antibiotics demonstrate quite high excretion rates, being approximately 90% for sulfamethoxazole, 65% for chlortetracycline, and, depending on the form of medication, between 50 and 100% for tylosin. Moreover, it is known that chlortetracycline strongly binds to soil particles and may accumulate in this environment, and that sulfamethoxazole persists in the environment for long periods and may be potentially washed off from the surface (Aust et al., 2008).

The most widely-used antibiotics in cattle production systems are tetracyclines, being used to treat skin, respiratory and gastrointestinal tract diseases. Furthermore, tetracycline resistance may develop rapidly because tetracycline ARGs are often located on MGE (Leclercq et al., 2016). In Europe, macrolides and lincosamides are also commonly used to treat a range of common infections in cattle such as mastitis, foot lesions, respiratory and genital infections. Many studies have examined the abundance of genes conferring resistance to MLSB (macrolides–lincosamides-streptogramin B) and tetracyclines in fecal microbial communities in beef cattle receiving antimicrobial agents, e.g., tylosin in fodder (Chen et al., 2008) (Table 2). Alexander et al. (2011) report the presence of five classes of tetracycline resistance genes in bovine fecal samples, as well as two classes of sulfonamide resistance genes and five classes of erythromycin resistance genes (Table 2).

**TABLE 2:** Antibiotic-resistant bacteria and antibiotic resistance genes in cow manure.

Bacteria species	Target antibiotics (major)	Resistance genes	Country	References
na	Macrolide (Erythromycin)	*ermB*, *ermF*, *ermT*, *ermX*	United States	Chen et al., 2008
	Tetracycline	*tetA/C*, *tetG*, and *tetM, tetO, tetP, tetQ, tetS, tetT, tetW*		
na	Tetracycline	*tetB, tetC, tetM*, *tetW*, *tetL*	Canada	Alexander et al., 2011
	Sulfonamide	*sul*1, *sul*2		
	Macrolide (Erythromycin)	*ermA*, *ermB*, *ermT*, *ermF*, *ermX*		
na	Beta-lactam	*bla2*	United States	Wichmann et al., 2014
	Aminoglycoside (Kanamycin)	*nat*, *aph*, *aacA-aphD*		
	Tetracycline	*tetW*, *tetO*		
	Chloramphenicol	*cat*		
na	Tetracycline	*tetC*, *tetM*, *tetW*	Canada	Holman et al., 2019
	Macrolide (Erythromycin)	*ermX*		
	Sulfonamide	*sul*2		
*Salmonella* spp.	Beta Lactam	*bla*_CMY–2_, *ampC*	United States	Winokur et al., 2001
*Escherichia coli*	Beta Lactam	*bla*_CMY–2_, *ampC*	United States	
	Beta Lactam	*bla*_TEM_, *bla*_CMY–2_, *bla*_CTX–M,_ *bla*_SHV_	Canada	Awosile et al., 2018
	Polymyxin (Colistin)	*mcr-1*	China	He et al., 2017b
	Beta Lactam	*bla*_VIM–2,_ *bla*_NDM–5_		
*Klebsiella* spp.	Beta Lactam	*bla*_TEM–1_, *bla*_SHV_*_–1_, bla*_OXA–1_	United States	Winokur et al., 2001
	Aminoglycosides	*rmtB, aac(6)-Ib-cr*		
	Quinoxalines	*oqxAB*		
	Quinolone	*qnrS1, qnrB2*		
	Beta Lactam	*bla*_NDM–5_	China	He et al., 2017a
	Beta Lactam	*bla*_KPC_, *bla*_SHV_, *bla*_TEM_	China	Yang et al., 2019
	Multidrug	*tol*C		
	Quinolone	*qnr*A, *qnr*B		
*Acinetobacter* spp.	Beta Lactam	*bla*_OXA–23_	France	Poirel et al., 2012
	Beta Lactam	*bla*_OXA–497_	USA	Webb et al., 2016
	Beta Lactam	*bla*_OXA–23,_ *bla*_OXA–58_	Lebanon	Al Bayssari et al., 2015
*Pseudomonas* spp.	Beta Lactam	*bla*_VIM–2_	Lebanon	Al Bayssari et al., 2015
*Enterococcus* spp.	Beta Lactam	*blaZ*	South Africa	Tanih et al., 2017
	Macrolides–lincosamides-streptogramin B	*ermB*		
	Tetracycline	*tetM*		
	Glicopeptide (Vancomycin)	*vanB*, *vanC1*		
	Glicopeptide (Vancomycin)	*vanC, vanA*	French	Haenni et al.,2009

*na, not applicable (data obtained from metagenomic study).*

The cattle industry also makes considerable use of β-lactams, first- and second-generation cephalosporins for mastitis treatment in dairy cattle. However, in the US, ceftiofur, a third-generation cephalosporin, is most commonly used for treating mastitis, respiratory disease, pododermatitis and metritis, while cefquinome, a fourth-generation cephalosporin, is applied for respiratory disease (Zalewska and Popowska, 2020).

Chambers et al. (2015) report that cows treated with ceftiofur demonstrate an increased proportion of β-lactam resistance and MDR in bacterial isolates compared to untreated controls, as well as a greater prevalence of gene sequences associated with phages, prophages, transposable elements and plasmids. These findings may suggest that treatment with this antibiotic may enhance ARG transfer. After ceftiofur treatment, additional functional shifts were noted, such as an increase in the proportion of gene sequences associated with stress response, chemotaxis and resistance to toxic compounds, and a decrease in those related to cell division, cell cycle and metabolism of aromatic compounds. In addition, measurable taxonomic shifts were observed, characterized by an increase in *Bacteroidia* and decrease in *Actinobacteria*. An increase in the number of *E. coli* isolates carrying *bla*_CMY–2_ was also observed during parenteral ceftiofur therapy; this can lead to a higher frequency of plasmid-mediated ARGs transfer by HGT to other enteric bacteria, including potential zoonotic pathogens. However, this would require not only the donor but also the recipient populations to be present in sufficient numbers (Stecher et al., 2012).

Although some previous studies have suggested that carbapenem-resistant bacteria are rare in livestock in the US (Webb et al., 2016; Mollenkopf et al., 2017), Vikram and Schmidt (2018) report the presence of a functional bla_KPC–2_ gene (*K. pneumoniae* carbapenemases) in the feces of beef cattle raised with antibiotics and those not; they also indicate that the *bla*_KPC–2_ gene may be mobilized. However, Agga et al. (2015) indicate that carbapenem-resistant genes are more frequently detected in effluent from US municipal wastewater treatment plant than in cattle catchment ponds for feedlot runoff or swine waste lagoons. These findings may suggest that carbapenem resistance may be more closely related to a close human environment than animal food production facilities.

Nevertheless, the dissemination of carbapenemase-producing bacteria in livestock has become a matter of global concern. Winokur et al. (2001) demonstrated the occurrence of *E. coli* and *Salmonella* spp. strains resistant to cephamycins and third-generation cephalosporins in feces samples isolated from food-producing animals. They also report the presence of carbapenemase-producing *Enterobacteriaceae* in dairy cow feces, e.g., *K. pneumoniae* harboring *bla*_NDM–5_, as well as various resistance genes including *bla*_TEM–1_, *bla*_SHV_*_–1_, bla*_OXA–1_, *rmtB, oqxAB, qnrS1, qnrB2, aac(6)-Ib-cr* (Table 2). In addition, carbapenemase-producing non-*Enterobacteriaceae* such as *Acinetobacter* spp. (related to *A. lwoffii*) harboring the *bla*_OXA–23_ carbapenemase gene have been isolated from dairy cattle feces, as have *Acinetobacter baumannii* with the *bla*_OXA–497_ gene (Bonardi and Pitino, 2019).

A study of rectal samples of dairy cattle in France by Poirel et al. (2012) identified the presence of *Acinetobacter* spp. isolates that were resistant to penicillins, combinations of penicillins with β-lactamase inhibitors, and carbapenems, but which were fully susceptible to cefotaxime. The identified bacteria also demonstrated reduced susceptibility to ceftazidime, and were resistant to tetracycline, kanamycin, and fosfomycin. The isolates with the *bla*_OXA–23_ gene were found to express β-lactamase OXA-23, which is widespread among *A. baumannii* (Table 2). Furthermore, the main vehicle for the *bla*_OXA–23_ gene among *Acinetobacter* spp. was identified as transposon Tn2008.

*Klebsiella pneumoniae* harboring the *bla_NDM–5_* gene was also isolated from samples of feces from dairy cows with mastitis in Jiangsu Province, China (Table 2). In all isolates, the *bla_NDM–5_* gene was found on an approximately 46 kb self-transmissible IncX3 pNDM-MGR194-like plasmid (He et al., 2017a). Additionally, He et al. (2017b) isolated and identified three NDM-5-producing *E. coli* isolates from dairy cows, including one co-producing the transferrable colistin resistance gene *mcr-1* and another co-harboring the carbapenemase gene *bla*_VIM–2_ (Table 2). The *bla*_NDM–5_- and *mcr-1*-harboring plasmids reduced the fitness of their bacterial hosts but maintained stability in the recipient strain. The *mcr-1*-carrying plasmid could be conjugated into NDM-5-positive *E. coli* isolates *in vitro*, thereby generating strains that eventually achieve pan resistance.

In addition, Al Bayssari et al. (2015) report the detection of VIM2-producing (*bla_VIM–2_*) *P. aeruginosa* and OXA-23-producing (*bla*_OXA–23_) *A. baumannii* in samples of cattle feces. They also identified the co-occurrence of *bla_OXA–23_* and *bla_OXA–58_* in the same isolate of *A. baumannii* (Table 2). The exact origins of these genes remain undefined, but they are known to have previously transferred from environmental bacteria into species with clinical relevance (Jiang et al., 2017; Waglechner and Wright, 2017). Woodford et al. (2014) note that the bla_OXA–48_ family of enzymes occurs naturally in *Shewanella* spp., a genus that inhabits lake sediments, and that OXA-23 carbapenemase originates from the environmental species *Acinetobacter radioresistens*, suggesting that these carbapenemase genes have probably been transferred from environmental bacteria into the animal microbiome, through the close contact between the host animal and the environment.

Importantly, commensal bacteria from animal intestines may serve as a reservoir of ARGs. *E. coli* is commonly used as an indicator species for monitoring AMR dynamics, especially for critical antimicrobials in veterinary and medicine, such as extended-spectrum cephalosporins (ESC) and fluoroquinolones (Reynolds et al., 2020). A number of studies have found *E. coli* strain O157 isolated from cattle feces from commercial and communal farms to be resistant to erythromycin, tetracycline, sulfamethoxazole, chloramphenicol, kanamycin, ampicillin and streptomycin (Ateba and Bezuidenhout, 2008; Volkova et al., 2012). Most infections caused by *E. coli* O157: H7 result from the consumption of food and water contaminated with the fecal matter of infected animals (Beauvais et al., 2018). Increased ESC resistance has frequently been reported in *E. coli* and *Salmonella enterica* from dairy cattle, and this was found to be caused mainly by ESBL or AmpC; many of these organisms have been classified as MDR (Sawant et al., 2007; Seiffert et al., 2013).

Kanwar et al. (2013) detected the presence of *tetA*, *tetB*, and *bla*_CMY–2_ genes in the feces of feedlot cattle; interestingly, the findings indicate that the use of chlortetracycline treatment following ceftiofur therapy could elevate the level of ceftiofur resistance. Jiang et al. (2006) report high resistance to ceftriaxone among various gram-positive and gram-negative bacteria isolated from calf feces.

Another important concern for human medicine is presented by the development of vancomycin resistance in bacterial strains with clinical significance such as *Enterococcus* spp. and *Staphylococcus* spp. Beukers et al. (2017) identified *E. faecium* and *E. faecalis*, which are potentially harmful to humans, in bovine feces; however, the Tn917 transposon conferring MLSB resistance was identified only in *E. faecium* and *E. hirae*. In addition, a study of cattle feces by Tanih et al. (2017) indicated the presence of four *Enterococcus* species, such as *E. hirae, E. faecium, E. durans*, and *E. faecalis* harboring tetracycline, erythromycin, penicillin, and vancomycin resistance genes (Table 2).

Jackson et al. (2011) examined AMR in ten different *Enterococcus* species, including *E. hirae*, *E. faecalis*, and *E. faecium*, in feces samples from US dairy cattle. In all species, the most prevalent resistance against an array of 17 antimicrobials was found to be against lincomycin, while all strains were resistant to at least one of the tested antimicrobials. Ten different types of AMR were observed among the *E. hirae* isolates, with one *E. hirae* isolate demonstrating MDR against seven antimicrobials. Interestingly, some *E. hirae* isolates were also resistant to one of the newest antimicrobials, daptomycin. In addition, *E. hirae*, *E. faecalis*, *E. faecium*, and another undetermined *Enterococcus* species demonstrated persistence to four antimicrobials (Jackson et al., 2011). Elsewhere, intrinsic vanC-mediated and acquired vanA-mediated resistance has also been reported in enterococci isolated from French cattle (Haenni et al., 2009) (Table 2).

Although pathogens resistant to only one antibiotic may cause severe infections that are very hard to treat, MDR bacteria, such as those isolated from cattle feces, are particularly dangerous. Yang et al. (2019) identified MDR in ESBL^+^ *K. pneumoniae* isolated from cow feces. The strain showed resistance to 13 antibiotics from almost all commonly-used antimicrobial classes. In addition, the following resistance genes were found: *bla*_KPC_, *bla*_SHV_, *bla*_TEM_, *qnr*A, *qnr*B, and *tol*C (Table 2). Most carbapenemases present in this *K. pneumoniae* strain belonged to the KPC group.

Studies conducted in Canada have shed light on the ARGs associated with ESC and fluoroquinolone resistance in *Enterobacteriaceae* in dairy calves. Awosile et al. (2018) identified *E. coli* isolates carrying *bla*_TEM_, *bla*_CMY–2_, *bla*_CTXM,_ and *bla*_SHV_ genes in the feces of dairy cattle (Table 2). In Germany, Klotz et al. (2019) isolated MDR strains from various non-fermenting bacilli, such as *A. baumannii*; these strains demonstrated resistance to ampicillin, amoxicillin-clavulanic acid, cefalexin, ceftiofur, nitrofurantoin, and chloramphenicol, with intermediate resistance observed for piperacillin (6%) and rifampicin (25%). Interestingly, the highest frequency of *A. baumannii* was observed in dairy cows, followed by beef cattle and calves.

Many studies focusing on the diversity of known and novel ARGs have been conducted. Wichmann et al. (2014) used a combination of functional metagenomics with PacBio sequencing to characterize the resistome of dairy cow manure. The prepared DNA libraries indicated the presence of genes coding resistance to β-lactams, kanamycin, tetracycline, and chloramphenicol (Table 2). In addition, the researchers identified 80 unique and functional ARGs, suggesting that the ARGs found in animal manure may originate from other genera such as *Proteobacteria*, *Firmicutes*, *Bacteroidetes*, and *Actinobacteria*. Hence, the full resistome appears to consist of phylogenetically varied organisms; in addition, many ARGs seem to be flanked by MGE such as transposases and insertion sequences, or may be created *de novo* like the newly-identified chloramphenicol resistance genes.

Research data indicates that even a single antimicrobial application can have a strong effect on the diversity and abundance of ARGs. Holman et al. (2019) found the fecal microbiota of beef cattle to be significantly altered on the second and fifth day after a single injection of either oxytetracycline or tulathromycin, and that they remained at this level until the 34th day after administration. Although five resistance genes (*ermX*, *sul*2, *tetC*, *tetM*, and *tetW*) were detected in the fecal microbiome, only *tetM* and *tetW* differed significantly between the animal groups divided according to the medicine administered: oxytetracycline application increased the prevalence of *tetM* in the fecal sample, while tulathromycin boosted the ratio of *tetM* to *tetW* resistance genes compared to animals receiving no antibiotic treatment. The presence of a single antibiotic in a bacteria-living niche may exert on-site selective pressure, thus facilitating the maintenance of ARGs, not only against this particular antimicrobial but also against unrelated resistance determinants through co-resistance; for example, co-transfer of *ermB* and *tetM* has been observed in the presence of erythromycin in *Streptococcus pyogenes* isolates (Brenciani et al., 2007). Fecal bacteria isolated from conventionally-raised cows, where antibiotics are added either as a growth promoter or for prophylaxis, tend to be more resistant to antibiotics than those from cows raised organically, i.e., where antibiotics application is forbidden or strongly supervised (Sato et al., 2005; Halbert et al., 2006); however, it seems that the observed increase in ARGs may be only temporary (Tragesser et al., 2006; Singer et al., 2008).

Interestingly, even cattle never exposed to antibiotics can also carry ARB and ARGs, such as a fluoroquinolone-, tetracycline- or β-lactam resistance genes (Durso et al., 2011). In addition, Thames et al. (2012) have detected genes coding resistance to tetracycline, sulfonamide, and macrolide in samples from newborn calves. In contrast, Roesch et al. (2006) found similar levels of antibiotic resistance between organic farms and conventional ones.

Reaching a common understanding that uncontrolled and unlimited usage of antibiotics may contribute to environmental pollution seems crucial to stopping the propagation of resistant bacteria in food animal production systems (Call et al., 2013).

## The Use of Antibiotics in the Pig Industry and Its Effect on the Swine Manure Resistome

Worldwide pig production has risen steadily during the last 50 years, reaching approximately 120 million tons in 2018. The leading producers of pig meat are China, with almost 45% of global production, followed by the US, Germany, Spain, and Vietnam. These five countries account for nearly 65% of world pig meat production (FAOSTAT, 2020).

An analysis of commercial pig feed formulation available in Vietnam has revealed that it may contain up to 55.4% antimicrobials, with the most common being bacitracin (24.8%), chlortetracycline (23.9%), and florfenicol (17.4%) (Van Cuong et al., 2016). It is estimated that 286.6 mg of in-feed antimicrobials are used to raise 1 kg of live pig (Van Cuong et al., 2016). Approximately 55.5% of antimicrobials administered to pigs are classified by the WHO as *important*, *very important*, or *critically important* for humans. A list of the antimicrobials intended for use in animals has been published by the OIE as a “list of antimicrobial agents of veterinary importance” (International Office of Epizootics, 2015).

One group of antibiotics considered as essential for human medicine is the polymyxins. Polymyxins are not routinely applied parenterally in animals because of their toxicity; however, one polymyxin, colistin, has been used extensively in pig production outside of North America as an oral treatment for neonatal colibacillosis (Dowling, 2013). In Southeastern Asia (India, China), colistin is used as a last-resort therapeutic option to treat severe infections caused by MDR gram-negative bacteria, including *P. aeruginosa* and *A. baumannii* (Catry et al., 2015). It is widely used in pig production for prophylactic (single animal), metaphylactic (whole pen or herd), and therapeutic purposes.

In addition, *mcr-1*, a plasmid-mediated colistin resistance gene, firstly identified in China (Liu et al., 2016), has also been found in Vietnamese pigs (Van Cuong et al., 2016). Colistin is also widely used in Europe (Catry et al., 2015; Kempf et al., 2016), and this may play a crucial role in the spread of colistin resistance in bacteria: although *mcr-1* has been identified in porcine fecal samples across Europe, it appears to be more predominant in Germany, Netherlands, Belgium, Denmark, Italy, Great Britain (Brauer et al., 2016; Guenther et al., 2017; Pulss et al., 2017), and Spain (Quesada et al., 2016), which may be due to differences in the regional regulations regarding its application. In addition, other variants of this gene have been found: *mcr-2* and *mcr-3* in China (Yin et al., 2017) and *mcr-2* in Germany (Roschanski et al., 2017).

Just as in cattle breeding, the use of ceftiofur and cefquinome in pig farming may be related to the spread of resistance to third and fourth generation cephalosporins among *Salmonella* spp. Resistance to ceftiofur in pigs was first identified in 2002 (*bla*_CMY_) (Hanson et al., 2002). A similar observation was also made in 2015 on Danish pig farms, where it was found that frequent use of third and fourth generation cephalosporins appeared to be related to the occurrence of ESBL producing *E. coli*. This resistance was also maintained over a prolonged period in animal herds (Andersen et al., 2015).

Some of the most extensively-studied ARGs are those conferring tetracycline resistance because this antimicrobial is widely administered to pigs. Research conducted on the hog population in the US in 2000 revealed a high level (71%) of tetracycline resistance in isolated *Enterococcus* spp. (Haack and Andrews, 2000). The gene *tetM* is known to confer the widest host range of tetracycline resistance and is often associated with MGEs, enhancing its transfer rate from one bacterium to another. More importantly, a number of tetracycline-resistant genes have been found not only in fecal swabs but also near swine feedlots. The genes found in pig fattening farms may code for a range of resistance mechanisms, such as efflux pumps, ribosomal protection peptides, or enzymatic modifications (Wu et al., 2010). The same genes have also been reported previously in swine lagoons, swine manure, storage pits, and groundwater located near pig production facilities (Table 3) (Chee-Sanford et al., 2001; Mackie et al., 2006; Koike et al., 2007). A study of the resistance profile of MDR *E. coli* from a pig farm and its surroundings in Germany (Table 3) identified similar ARGs in samples taken from stable flies and from the feces of barn dogs, indicating the potential for AMR to spread across the surrounding environment (Guenther et al., 2017).

**TABLE 3:** Antibiotic resistance genes in pig.

Resistance genes	Strain	Source	County	References
*mcr-1*	*E. coli*	Intensive pig farm	China	Liu et al., 2016
	na	Pig	Vietnam	Van Cuong et al., 2016
		Porcine fecal samples	Germany Netherlands Belgium Denmark Italy Great Britain	Brauer et al., 2016; Guenther et al., 2017; Pulss et al., 2017
*mcr-2*	*E. coli*	Porcine fecal sample	China	Yin et al., 2017
		Pooled feces and boot swab samples	Germany	Roschanski et al., 2017
*mcr-3*	*E. coli*	Porcine fecal sample	China	Yin et al., 2017
*tetA, tetC, tetE, tetG, tetK, tetL, tetA/P, tetM, tetO, tetQ, tetS, tetT, tetW, tetB/P, tetX*	na	Porcine feces swine lagoons manure storage pits groundwater near pig production facilities		Chee-Sanford et al., 2001; Mackie et al., 2006; Koike et al., 2007; Wu et al., 2010
*aadA5*, *aph(3′)-Ic-like*, *bla*_CTX–M–1_, *dfrA17*, *strA*, *strB*, *sul2-like*, *tet(B), aadA1, bla_TEM–1B_, dfrA1, mcr-1, sul1, sul2, tetA, aadA2, cmlA1-like, mph(A), sul3, tet(A)-like*	*E. coli*	Pig manure	Germany	Guenther et al., 2017
*bla_TEM–1B_-like*, *strA-like*, *strB-like*, *tetA, aadA1, aadA2, aph(3′)-Ia-like, bla_TEM–1B_, cmlA1-like, dfrA1, mcr-1, sul3*		Animal boot swabs		
*aadA1*, *bla*_TEM–1B_, *dfrA1*, *dfrA14-like*, *mcr-1*, *strA*, *strB*, *sul1*, *sul2*, *tet(A)*		Stable fly		
*bla_TEM–1B_-like*, *dfrA1-like*, *mcr-1*, *strA-like*, *strB-like*, *tetA*		Barn dog feces		
*bla_CTX–M,_ ermB, mcr-1, optrA, qnrS, tet(40), aac(6′)-lm*	na	Pig feces	Belgium, Bulgaria, Germany, Denmark, Spain, France, Italy, Netherlands, Poland	Munk et al., 2018
*mexF*, *oprD, aadA2*, *aadD*, *aacA/adhD*, *aphA1, fox5, tetPB*, *tetQ*, *tet(32)*, *tetL*, *tetO, ermT*, *ermX*, *lnuB*, *vatE, sat4*	na	Pig feces		Zhao et al., 2018
*tet36*, *tetM*, *tetQ tetX*, *tetP(A), tet40, ermF*, *mefA, sul1*, *sul2, aac(6)-le*, *aph(6)-ld, catB3*, *catA16, oxa9*, *cfxa, mexD*	na	Swine fattening facility surroundings (well water, swine wastewater, soil, fishpond)	China	He et al., 2019
*sul1, tet(32), tetL, tetM, tetO, tetBP-03, tetQ, tetW, tetX, floR, mexF, aadA, aadA1, aadA2, aadD, aph(2′)-Id, aphA3, ermA, ermB, vatE, sat4*	na	Pig slurry	China	Pu et al., 2018
*bla*_OXA–48_, *bla*_TEM–1B_, *aph(3′)-Ia*, *aadA1*, *aadA2*, *aac(3)-IId*, *Inu*(F), *qnrS1*, *floR*, *cmlA1*, *sul2*, *sul3*, *tet*(A), *tet*(M), *dfrA12, aph(4)-Ia, aadA5, aac(3)-IVa, armA, dfrA17*	*E. coli* pathovars	Porcine fecal samples	Germany, Netherlands Belgium Denmark Italy Great Britain	Pulss et al., 2017
*tetM*, *tetO, bla*_TEM–1,_ *bla*_SHV–2_, sul2, *aph(3′′)-Ib*, *aph(6′)-Ic*, *aph(6′)-Ib, ermD*, *mdfA*, *mdtH*, *mdtL*, *rosA*, *tet(B), bcr, adeA*, *armB*, *mdtF, mdtN, mdtO, mdtP, oprA, tolC, acrA*	na	Porcine feces samples	The US	Looft et al., 2012

*na, not applicable – dataset was obtained during metagenomic study.*

A study of the resistomes of non-typhoidal *Salmonella* strains isolated from swine fecal samples found the highest levels of AMR to be against tetracycline (80% of isolates), streptomycin (43.4%), and sulfamethoxazole (36%). The results also indicated that the microbes isolated from farms raising animals with antimicrobials tended to demonstrate greater resistance to most classes of antibiotics than antimicrobial-free farms (Gebreyes et al., 2006). Furthermore, MDR *Campylobacter coli* have been found within pig production systems (Thakur and Gebreyes, 2005).

More than 400 ARGs were identified in a study conducted in nine European countries (Belgium, Bulgaria, Germany, Denmark, Spain, France, Italy, Netherlands, and Poland) on 181 pig herds and 178 poultry farms. These studies indicated that fecal pig resistome [*bla_CTX–M_, ermB, mcr-1, optrA, qnrS, tet(40), aac(6′)-lm*] differs significantly between countries. This discovery appears to be closely related to the antimicrobial usage policy of each country: countries with a strict policy tend to have a lower rate of antibiotic resistance. Conversely, pig herds located in the same country or countries with a similar antimicrobial usage policy tend to demonstrate similar ARGs patterns. The newly-identified *optrA* gene (enterococcal linezolid resistance group), which codes resistance to both oxazolidinone and amphenicols (florfenicol), was detected in countries with the highest usage of amphenicols, such as Bulgaria, Italy, and Spain (Munk et al., 2018).

Zhao et al. (2018) identified a total number of 146 ARGs in animal feces from seven large-scale Chinese pig farms, i.e., two with high levels of medication and five with low levels, including eight transposon-transposase genes and two class 1 integron-integrase genes. These ARGs genes represented all major classes of resistance mechanisms: 44.5% conferring antibiotic deactivation, 34.3% coding for efflux pumps, and 19.2% cellular protection. These mechanisms provide resistance for all major antimicrobial classes: aminoglycoside (*aadA2*, *aadD*, *aacA/adhD*, *aphA1* [aka *kanR*] – 20%), β-lactam (*fox5* – 14%), tetracycline [*tetPB*, *tetQ*, *tet(32)*, *tetL*, *tetO* -14%], MLSB (*ermT*, *ermX*, *lnuB*, *vatE –* 14%), vancomycin (8%), sulfonamide (2%), chloramphenicol (2%), and others (*sat4* – 4%). The copy number of the gene ranges from 2.72 × 10^9^ to 1.34 × 10^10^, divided mostly between tetracycline (4.77 × 10^9^), MLSB (3.41 × 10^9^), and aminoglycoside (4.02 × 10^9^) ARGs (Zhao et al., 2018).

A wide variety of ARGs have also been found in wastewater from pig farms. Of these, tetracycline resistance genes predominated, followed by MLSB resistance and sulfonamide resistance, with aminoglycoside resistance, phenicol resistance, and β-lactam resistance being slightly less abundant (Table 3) (He et al., 2019). In addition, the initially identified genes were also identified in pig wastewater-irrigated soils; however, their type and relative abundance varied depending on the sampling depth.

Another analysis conducted in China in 2018 identified 21 ARGs and three transposons (Table 3) in pig slurry originating from a high production livestock facility. The ARGs were divided into four resistance mechanisms: cellular protection (43%), antibiotic deactivation (38%), efflux pump (14%), and others (5%). They thus conferred resistance to seven major classes of antibiotics: tetracycline (38%), florfenicol-chloramphenicol-amphenicol (29%), MLSB (14%), aminoglycoside (9%), sulfonamide (5%), and others (5%) (Pu et al., 2018).

A study conducted by Pulss et al. (2017) also detected many other ARGs in an *E. coli* strain carrying an OXA-181 carbapenemase (Table 3).

An analysis of the feces from animals supplemented with typical performance-enhancing antibiotics (chlortetracycline, sulfamethazine, and penicillin) in the US revealed increased abundance and diversity of ARGs in the gut microbiome compared with animals raised without antibiotics. Six types of resistance mechanisms were found to be more abundant in the treated animals: those coding for tetracycline efflux pumps, class A β-lactamases, sulfonamide resistance genes, aminoglycoside *O*-phosphotransferase, and two types of multidrug efflux: a major facilitator superfamily transporter, and a resistance-nodulation-cell division transporter system (Table 3). However, some genes were also detected at high frequencies in non-medicated pigs. It is possible that their presence may reflect antibiotic resistance developed by farm animals due to selection pressure exerted by over 50 years of antimicrobial treatment (Looft et al., 2012).

Additionally, heavy metals such as zinc, copper, lead, cadmium, chrome, and nickel have been commonly found in animal manure. It has been proven that high doses of copper and zinc enhance the growth of animals (Poole, 2017), and it is possible that these were supplied as mineral addition to fodder; however, others, such as lead, cadmium, chrome or nickel, may have originated in corroded metal parts of installations (Zhao et al., 2018). It is known that heavy metal tolerance is linked to AMR: it may select for resistant bacteria and facilitate the persistence of resistance genes by cross-resistance. It has also been proven that the presence of sublethal doses of heavy metals in the environment facilitates the conjugative transfer of ARGs (Zhang et al., 2018).

The use of copper and zinc in pig fodder has been found to be closely associated with the presence of MDR *Salmonella* spp. (Barton, 2014). Compared to copper-sensitive strains, copper-resistant strains are more likely to be resistant to ampicillin, tetracycline, chloramphenicol, or sulfonamides, as well as to penicillin, just as nickel-resistant strains are also resistant to ampicillin. Moreover, lead resistance may be linked to resistance to β-lactams, mercury tolerance to streptomycin resistance, or lead and mercury tolerance to ampicillin resistance (Sabry et al., 1997; Bass et al., 1999; Berg et al., 2010). Such cross-resistance between heavy metals and antibiotic resistance in the pig production chain has been confirmed by Hölzel et al. (2012).

An interesting example of a plasmid carrying both ARGs and metal tolerance genes was described by Fang et al. (2016). One of the most common incompatibility groups of plasmids in the *Enterobacteriaceae* is IncHI2. It has been identified in *E. coli*, *S. enterica*, *K. pneumoniae*, and *Enterobacter cloacae* isolated from humans, chickens, and rarely swine. The plasmid may harbor a number of metal tolerance genes, such as *pcoABCDRSE* (efflux systems to detoxify copper), *silESRCBAP* (efflux systems to detoxify silver), ars*CBRH* (efflux systems to detoxify arsenic), *merEDACPTR* (Tn1696-related mercury operon), and *terZABCDEF* and *terY3Y2XY1W* (tellurite resistance systems). It also contains a number of AGRs coding resistance to amphenicols (*floR)*, aminoglycosides (*armA*, *aac-Ib*/*aac-Ib-cr*), β-lactams (*bla*_CTX–M_, *bla*_CMY_, *bla*_SHV_, *bla*_*IMP*_, *bla*_VIM_), quinolones (*oqxAB*, *qnrA1*, *qnrS1* and *qnrB2*), and fosfomycin (*fosA3*). A plasmid harboring both ARGs and metal tolerance genes, Sal-1457, has been identified in *Salmonella infantis* isolates obtained from the feces and carcasses of goats, cattle and chickens in Australia; it has been found to contain *aph(3″)-I*, *aph(6″)-ld*, *bla_TEM–1_*, and *dfrA5*, together with an arsenic resistance operon (Wilson et al., 2019). A very detailed study found almost 17% of analyzed bacterial genomes in samples obtained from multiple environments to carry both ARGs and metal tolerance genes (Pal et al., 2015).

## Changes in the Soil Resistome Occurring Due to Manure Application

The soil has been identified as a critical source of ARGs, not only because of the presence of a diverse range of bacteria able to produce natural antibiotics (Lang et al., 2010) but mainly because of the application of natural manure on crop fields, which might contain ARGs or antibiotics: only a small amount of antibiotics are absorbed or metabolized by animals, with about 75% of the administered drug being excreted into the feces or urine (Zhang and Zhang, 2011).

Soil fertilization with animal manure is a widespread agricultural practice, not only in Europe but worldwide, especially in less developed countries (Heuer et al., 2011). This kind of manure is known to be a rich source of nutrients and organic matter for fertilizing fields. The use of organic manure as fertilizer can also be a practical approach to animal waste management by lowering the cost of its disposal: a single dairy cow can produce 54 kg of wet manure per day, a pig – 6.4 kg, a sheep – 2.5 kg, and a chicken – 0.2 kg (Girotto and Cossu, 2017). Furthermore, fertilizing arable soil with manure can play an essential role in the active cycling of chemicals which are crucial for optimal crop growth and development, such as phosphorus or nitrogen. However, besides its doubtless advantages, the misuse or overuse of manure field applications may account for the excessive amount of these elements in soils and the accumulation of heavy metals added to feeders.

Antibiotics can react and accumulate in specific ways following manure application depending on physicochemical properties of soil and climate conditions. A study of columns filled with types of soil by Pan and Chu (2017) found that the leaching of antibiotics is generally higher in sandy soil than in clay and silty soil. Antibiotics such as norfloxacin and tetracycline were also found to persist longer at the soil surface, contrary to sulfamethazine and erythromycin that tend to reach deeper layers of soil and groundwater.

The full biotransformation and degradation of antimicrobials may take up to 150 days in bovine manure (De Liguoro et al., 2003). Although manure is an essential and sufficient source of nitrogen for agricultural soil, it is also a rich source of antibiotics and ARB, which may be transferred to the environment and survive there even for several months (Merchant et al., 2012). Therefore, it is extremely important to develop effective treatment strategies for manure used as natural fertilizer, with the aim of eliminating, or at least reducing, the risk of releasing antibiotics, ARGs, and ARB to the environment (Zalewska and Popowska, 2020).

Some antibiotics with high adsorption capacity, such as tetracyclines and quinolones, can actively adhere to soil particles, making them non-biodegradable. They can thus accumulate in agricultural soil amended with manure, changing the natural microbial community structure and promoting the maintenance of resistance genes (Li et al., 2011). There are several possible explanations for the increasing abundance of ARGs observed in manured soils. Firstly, it is possible that, following manure application, any ARGs present in bacteria that will survive in the environment can be simply transferred to new hosts by HGT. Alternatively, residues of active antibiotic compounds in manure can induce new mutations in soil bacteria, or enrich and sustain pre-existing ones. Lastly, animal waste rich in organic matter can enhance the growth of resistant resident bacteria in soil (Udikovic-Kolic et al., 2014; Xie et al., 2018). In addition, heavy metals present in soil act as major stress factors in the environment, and as antibiotics and heavy metals can share the same regulatory responses, their presence may also promote ARG selection *via* co-selection and cross-resistance mechanisms (Imran et al., 2019).

A study in Shandong Province, China examined the abundance of ARB and ARGs in farmland soil fertilized with chicken manure (Zhao et al., 2017). The results indicate the presence of a number of ARB and ten AGRs, such as *tetW, tetO, tetT, tetM, tetA, tetL, tetQ, sul1, sul2*, and *sul3*, in soil samples collected from four fields. In all samples, researchers observed significant correlations between the concentration of applied sulfonamides and the abundance of sulfonamide resistance genes.

Similar studies from Finland examined changes in ARGs and MGE abundance in the soil after fertilization with swine and cow manure. The results identified the presence of ARGs, previously detected in animal waste, in fertilized soil. However, while the abundance of ARGs associated with manure generally decreased following 2 and 6 weeks after fertilization, the levels of some ARGs, such as those conferring resistance to disinfectants, aminoglycosides, and vancomycin, remained elevated compared to unfertilized soil, even after 6 weeks (Muurinen et al., 2017). In addition, Marti et al. (2014) report greater abundance of selected genes related to AMR and gene transfer, such as *sul1*, *ermB, strB*, *int1*, and *repA*, in soils fertilized with cow and swine manure than non-manured soil.

Studies also indicate that the increase in ARB observed in soil treated with cow manure may be due to the bloom of resistant species already present in the soil, rather than resistant bacteria introduced from the manure itself (Udikovic-Kolic et al., 2014). The manure used in this study was obtained from dairy cows that had not been treated with antibiotics.

The length of time for which ARG levels remain elevated varies considerably depends on various factors, such as weather conditions or soil type. Increased levels of *sul1* and *sul2* genes were observed for over 4 months following swine manure application in field plots in Germany (Jechalke et al., 2014), while *intI1* persisted in soil for 10 months after swine manure application in agricultural fields in the United Kingdom (Byrne-Bailey et al., 2011). Tighter regulations regarding manure application are in force in the US: manure application must be performed no less than 120 days before harvesting crops that have direct contact with manured soil and no less than 90 days for crops with no direct contact with the soil (Ferguson and Ziegler, 2004).

## Changes Of The Resistome In Plants Growing On Manure-Fertilized Soil

Fruits and vegetables often harbor non-pathogenic epiphytic microflora; however, many studies have reported contamination with pathogens or different types of ARGs; such contamination may occur pre-harvest *via* soil and organic fertilizers such as manure, sewage sludge, and irrigation water. This is a serious consideration as any contaminated vegetables intended to be consumed raw may act as vehicles for the spread of ARB and ARGs to humans (Hölzel et al., 2018).

Sub-inhibitory concentrations of antibiotics in plant tissues have been found to be potential drivers of antibiotic resistance in endophytic bacteria, and small amounts of antibiotics, such as tetracycline, can trigger HGT between different bacteria (Redondo-Salvo et al., 2020).

Antibiotic resistance genes can also be transferred to plants from common soil bacteria *via* root endophytes, and these have been found to survive in the root (Bulgarelli et al., 2012, 2013; Lundberg et al., 2012). Some endophytes appear to be are closely related to human pathogens, particularly opportunistic ones (Rosenblueth and Martínez-Romero, 2006), and this similarity could pose a potential threat to human health. Solomon et al. (2002) demonstrated the transmission of *E. coli* O157: H7 from manure-contaminated soil and irrigation water to lettuce; it is believed that these bacteria can enter the lettuce through the root system and propagate throughout the edible portion of the plant.

The soil may well represent the main source of ARGs to the plant, as indicted by the large degree of overlap between ARGs in the plant microbiome and those in the soil resistome (Yang et al., 2018). ARB associated with soil and manure may enter the plant microbiome by colonizing the roots, which are in direct contact with soil, or the aboveground parts, potentially through air particulates or the motility of root endophytes (Zhu et al., 2017; Guron et al., 2019). In addition, plants have been found to take up antibiotic residues from manure-amended soil; this may exert long-term pressure in the plant, facilitating the acquisition of drug resistance and its spread across the plant resistome (Chen et al., 2019a).

The phyllosphere, i.e., aerial leaf surfaces, is a specific niche harboring diverse species of bacteria and other microbes (Bulgarelli et al., 2013). Many previous studies have examined the impact of manure application on the levels of ARGs in the phyllosphere of leafy vegetables (Chen et al., 2016, 2017), or changes in ARGs abundance and dissemination (Marti et al., 2013; Tien et al., 2017; Murray et al., 2019). MGE such as *int*I1 and genes encoding transposase have been detected in leaf endophytes, as well as in the phyllosphere of lettuce (Wang et al., 2015; Zhu et al., 2017), maize (Chen et al., 2016), *Brassica chinensis* L (Chen et al., 2019a), and *Coriandrum sativum* L (Chen et al., 2019b).

After excretion by animals, pollutants such as antibiotics, antibiotic residues, ARB and ARGs may be transported through the environment *via* runoff, leaching and manure application (McEachran et al., 2015). These can accumulate in soil and increase the risk of selection pressure and crop contamination with ARB and ARGs (Hu et al., 2010). The ability of plants to take up antibiotics depends on several biotic and abiotic factors, as well as the type of crop: fruit and grain demonstrate a lower ability to absorb contaminants than leafy and root vegetables (Christou et al., 2019).

Manure application has been found to increase the abundance of ARB and ARGs in soil (Udikovic-Kolic et al., 2014; McKinney et al., 2018). In addition, manure-amended soils have been associated with increased detection of ARB and ARGs on lettuce and root vegetables; however, this has not been associated with all crops or ARGs (Marti et al., 2013; Rahube et al., 2014; Wang et al., 2015). It is important to note that while fecal bacteria can survive for weeks to months in the environment, depending on species and temperature, genetic elements can persist for much longer, regardless of cell viability (Chee-Sanford et al., 2009). ARB, which are naturally found in manure, may attach to the crops grown in the amended soil and multiply in this new potentially more favorable environment. As a consequence, ARB can become dominant among the natural resident bacterial population (Baquero, 2011; Wichmann et al., 2014).

Humans can become exposed to bacteria carrying ARGs and various pathogens by consuming contaminated vegetables (Berger et al., 2010; van Hoek et al., 2015). Yang et al. (2014) examined the effect of soil amendment with chicken manure on the distribution of antibiotic resistant endophytic bacteria (AREB) and MDR endophytic bacteria in celery, pak choi and cucumber. High numbers of bacteria resistant to at least one antibiotic were identified in all vegetables; however, MDR bacteria were found to be absent from the surface of the tissue or inside the vegetables. However, Yang et al. (2016) report that the application of chicken manure or organic fertilizer increases the populations of MDR bacteria in soil and MDR endophytic bacteria in pak choi. Yang et al. (2014) found the highest amounts of cultivable endophytic ARB in celery roots. This is not surprising, as in most plants, roots generally have the highest rates of total cultivable AREB, and hence may serve as a reservoir for potentially pathogenic bacteria or ARB (Berg et al., 2005).

A study of ARB in wheat by Yang et al. (2009) found that AREB were most prevalent in the roots, followed by the leaves, with no AREB detected in the seeds. A report by Sengeløv et al. (2003) indicated that the occurrence of tetracycline-resistant bacteria in the soil was elevated after pig manure slurry treatment, but this fell to control values over the following 5 months. Furthermore, Schmitt et al. (2006) found that both pig manure and the amended soil contain a wide variety of tetracycline ARGs following fertilization. A number of other publications indicate that ARGs can infiltrate the edible parts of commercial crops and that their presence and concentrations depend upon farming practices (Manaia et al., 2018; Cerqueira et al., 2019a, b).

Marti et al. (2013) evaluated the occurrence of ARB on the surface of vegetables that are often eaten raw, such as tomatoes, cucumbers, peppers, carrots, radishes and lettuce, and compared their presence in vegetables grown in inorganically-fertilized soil with those in soil fertilized with dairy or swine manure. The selected vegetables included a range of roots, fruits, and leafy vegetables with different degrees of presentation to key environmental factors (e.g., sun, rain, or wind). It was found that fertilization only had a significant effect on the abundance of amoxicillin-clavulanic acid, ampicillin, and cefoxitin resistance genes. Interestingly, in the case of carrots, a reduction in the abundance of ampicillin- or cefoxitin-resistant bacteria was observed compared to the control group. Across the sample as a whole, eight gene targets (*sul*2, *tet*B, *tet*T, *erm*A, *erm*F, *qnr*B, *bla*_*PSE*_, and *bla*_OXA–20_) were detected on at least one vegetable sample. Plants grown in dairy manure-amended soil carried *sul2*, *erm*F, *qnr*B, *bla*_*PSE*_, and *bla*_OXA–20_, while those grown on swine manure-amended soil harbored *sul2*, *tet*B, *tet*T, *erm*A, *erm*F, and *bla*_OXA–20_ (Marti et al., 2013).

Cerqueira et al. (2019b) examined the distribution of ARGs in soil, rhizospheric soil, roots, and leaves for tomato, lettuce, and broad beans grown in nine commercial plots. All plots had been fertilized with manure obtained from fairly unusual sources such as horse or pigeon feces. Of the ARGs, the most prevalent was *bla*_TEM_, which was detected in all analyzed samples. The data showed that ARGs loads decreased gradually from soil to fruit, resulting in a 100- to 1000-fold dilution for most studied genes; in addition, the ARGs concentration and composition of edible plant parts was strongly dependent on the initial soil ARGs content (Cerqueira et al., 2019b).

Fogler et al. (2019) found the compositions of microbiota and resistomes associated with vegetable surfaces to be strongly influenced by the amendment of soil with raw manure collected from dairy cows during antibiotic administration, especially clindamycin. Their findings indicate that the genes *tetW* and *sul*1 are ubiquitous and present in high abundance in the commensal bacteria inhabiting humans and domestic animals, and that they demonstrate a consequently high representation in waste streams, a low abundance in less-affected environments and a uniform and a highly-conserved DNA sequence; they may therefore serve as valuable markers of soil contamination with ARB, ARGs, and antibiotic residues originating from manure. In addition, lettuce grown in manure-amended soil demonstrated a more diverse bacterial composition than lettuce grown in soil supplied with chemical fertilizers. No significant differences were observed between the relative abundances of detected bacterial phyla/classes based on the type of amendment (Fogler et al., 2019).

Wang et al. (2015) identified ten tetracycline resistance genes (*tetA, tetP, tetC, tetG, tetL, tetB, tetM, tetO, tetW, tetX*,) and two sulfonamide resistance genes (*sul1* and *sul2*), along with one *int1* gene, in root endophytes, leaf endophytes, and phyllosphere microorganisms from vegetable samples grown in manure-amended soil. The prevalence of ARGs was generally lower in leaf endophytes than in the root or phyllosphere microorganisms. Interestingly, it was observed that, for lettuce and endive, the occurrence of ARGs, such as *sul*1, *sul*2, *tetC*, and *tetG*, in root endophytes, leaf endophytes and the phyllosphere members, was also affected by growth period and species of plant (Wang et al., 2015).

A considerable number of studies have reported the presence of significant numbers of ARB on consumer-ready vegetables, such as lettuce and spinach leaves (Abriouel et al., 2008; Bezanson et al., 2008). Zhang et al. (2020) constructed a pot experiment to determine the effect of poultry and cattle manure application on resistome shifts in the plant microbiome of harvested cherry radish. A total of 144 ARGs conferring resistance to eight major classes of antibiotics were detected across all samples. The abundance of MGE was found to be positively correlated with individual ARGs coding for resistance to aminoglycoside, fluoroquinolone, quinolone, florfenicol-chloramphenicol-amphenicol, sulfonamide and tetracycline, and MDR determinants including MLSB. The results suggest that manure application significantly increased the abundance of ARGs in the rhizosphere and phyllosphere but not in the endophytes of the root, i.e., the edible part (Zhang et al., 2020).

The abundance of two MGEs (intI and tnpA-05) also appears to significantly increase after poultry manure application, indicating that not only does manure application directly introduce ARGs to the soil, but also may increase the HGT rate for ARGs; it also suggests that the transmission of ARGs from manure/soil to the surface of vegetables may be the predominant dissemination route. However, although the ARB involved in the transfer colonize as root endophytes, they demonstrate very little transfer to plant tissues; this suggests that the risk of transmitting external ARGs to the food chain is low. Therefore, root vegetables like cherry radish might be at a lower risk of ARGs contamination than leafy vegetables like lettuce (Zhang et al., 2019, 2020).

The degree to which livestock and agricultural land act as reservoirs of antibiotic-resistant bacteria, and how these two factors interact are relatively unknown (Tyrrell et al., 2019). Many studies have attempted to trace the direction of gene transfer from the environment to manure and determine its implications for future antibiotic resistance management and microbial ecology (Cook et al., 2014; Nesme and Simonet, 2015). In addition, evidence suggests that the primary pathway of gene acquisition from different environments may be HGT, including transfer from soils to the genomes of pathogenic bacteria (Allen et al., 2009; Forsberg et al., 2012; Nesme et al., 2014). Furthermore, DNA element class 1 integrons allow bacteria to adapt and evolve by integrating foreign genes from the environment through the capture of MGEs. This phenomenon has played an essential role in spreading AMR from non-pathogenic bacteria to pathogenic bacteria in the environment (Zhu et al., 2017).

## Concluding Remarks

**FIGURE 3:**
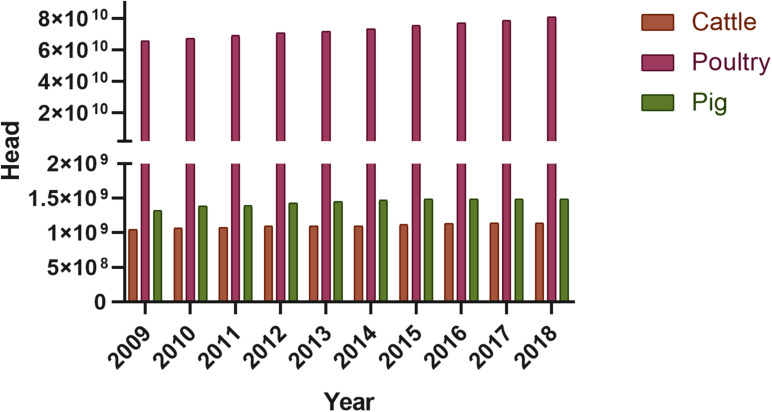
Animal production worldwide (according to FAOSTAT, 2020).

Although many promising solutions aimed at the reduction of bacterial resistance and overuse of antibiotics in the fields of animal production have emerged (Allen et al., 2013; Cheng et al., 2014; Tabashsum and Biswas, 2019), it seems that no single approach will be able to replace all the antibiotic applications in the animal production sector. Fortunately, public awareness is growing of the harmful effect of antibiotic usage on farm animals, and with it consumer demand for food products with guaranteed quality obtained from animals raised humanely and with minimal environmental impact. However, the demand for animal-derived food products is increasing with rising global population and economic growth (Figure 3). This global trend is well illustrated in the FAOSTAT database. Hence, the combination of steadily growing consumption with lack of regulation or intervention strategies, particularly in developing and middle-income countries, will no doubt result in increasing levels of antimicrobial usage, mostly due to the shift from extensive farming to large-scale production systems (Van Boeckel et al., 2015). It is assumed that the global consumption of antimicrobials in livestock and humans will increase by 67% between 2010 (63,151 tons ± 1560) and 2030 (105,596 ± 3605), of which approximately two-thirds will be associated with the rising number of food-producing animals (Bloomer and McKee, 2018).

The relationship between antimicrobial use in farm animals and spread of ARB/ARGs is undeniable, as is the risk of their spread to pathogens posing a threat to human health. Antibiotic resistance should be considered a global threat, because neither bacteria nor genes respect geographical or national obstacles.

The most direct way to restrain the spread of AMR is to reduce or optimize their application in animal farming practices. The first step is to improve animal keeping conditions and herd management systems: animals remain healthy when they receive good quality, well-balanced fodder, and are kept in facilities with exceptional hygiene. Moreover, a range of prevention strategies such as vaccination, or the addition of probiotics, prebiotics, or bioactive compounds (e.g., antimicrobial peptides) in fodder can be used to protect vast herds from infection, and subsequently limit antimicrobial usage. In addition, guidelines should be prepared for good practice regarding livestock waste, such as manure management or wastewater treatment strategies (Zhao et al., 2020).

## Author Contributions

MZ and MP: conceptualization and writing-review and editing. MZ, AB, AC, and MP: writing – original draft preparation. MZ, AB, and AC: visualization. MP: supervision, project administration, and funding acquisition. All authors read and agreed to the published version of the manuscript.

## Conflict of Interest

The authors declare that the research was conducted in the absence of any commercial or financial relationships that could be construed as a potential conflict of interest.
